# Reimagining Paediatric HIV Care: Centering Infants and Young Children in Optimized, Integrated Models of Care

**DOI:** 10.1002/jia2.70103

**Published:** 2026-06-19

**Authors:** Fatima Tsiouris, Catharina Alons, Madeline Maurice, Nilesh Bhatt, Natella Rakhmanina, Michelle M. Gill, Anja Giphart, Appolinaire Tiam

**Affiliations:** ^1^ Elizabeth Glaser Pediatric AIDS Foundation Washington DC USA

**Keywords:** HIV models of care, HIV service delivery, HIV, paediatric tailored, retention in care, viral suppression

## Abstract

**Introduction:**

Despite substantial gains in the global HIV response, infants and young children continue to experience poorer outcomes across the HIV testing and care cascade. Rates of early infant diagnosis, timely antiretroviral therapy (ART) initiation, retention in care and viral suppression remain unacceptably low in paediatric populations. These disparities are compounded by systemic challenges, including fragmented service delivery, limited availability of child‐friendly ART and other drug formulations, and health systems that fail to integrate HIV services within broader maternal and child health platforms. As a result, many children living with HIV are diagnosed and start treatment late and face high risks of mortality and morbidity, particularly in the first 2 years of life.

**Discussion:**

In this commentary, we advocate for the urgent reimagining of paediatric HIV service delivery, emphasizing the need to centre infants and young children within models of care. We highlight four promising approaches: (1) Integrated mother−infant follow‐up models that ensure continuity of care from pregnancy through the postpartum period and infancy until the infant's final HIV status is determined; (2) Family‐centred models that treat the household as the unit of care; (3) Advanced HIV care strategies tailored to the needs of children with late presentation or treatment failure, including paediatric tailored ART regimens and diagnostics; and (4) Community‐based interventions that leverage peer support, lay health workers, and provide stigma‐free entry points to expand access and retention to care and treatment. These approaches have demonstrated that when services are designed to reflect the developmental, clinical and social needs of children and their caregivers, outcomes significantly improve. However, many of these models remain underutilized, fragmented or inadequately resourced.

**Conclusions:**

To close the paediatric HIV treatment gap, we must move beyond pilot projects towards national, integrated and adequately funded child‐centred systems. This requires political will, strategic investment and prioritization of children in HIV policy, innovation and implementation research. A sustained and equitable HIV response must include infants and young children not as an afterthought, but as a core priority. Epidemic control cannot be achieved if the youngest children are left behind.

**Abbreviations**
AHD: advanced HIV disease
ART: antiretroviral therapy
CDIs: community‐driven interventions
CLHIV: children living with HIV
CrAg: cryptococcal antigen
DTG: dolutegravir
EID: early infant diagnosis
FCCM: family‐centred care model
HIV: human immunodeficiency virus
HIVST: HIV self‐testing
MMD: multi‐month ART dispensing
PrEP: pre‐exposure prophylaxis
PVT: prevention of vertical transmission

## 1 Introduction

Although the global HIV response has seen significant progress, children living with HIV (CLHIV) continue to experience disproportionately poor outcomes across the HIV care cascade. Early diagnosis, timely initiation of antiretroviral therapy (ART), sustained viral load suppression, and long‐term retention in care and treatment remain elusive for far too many children. Paediatric HIV services, especially for children under 5, lag in visibility, investment or innovation.

Globally, an estimated 1.4 million children are living with HIV, yet only 55% are currently on treatment [1]. Many children are diagnosed late, only after presenting with advanced HIV or opportunistic infections, reflecting systemic failures in early infant diagnosis (EID) and engagement and continuity in care. These delays are further exacerbated by fragmented service delivery systems or limited child‐friendly approaches along the care continuum. The paediatric HIV response has faced struggles, in part because current service delivery models fail to reflect the realities of the lives of children and their caregivers. Infants and young children depend on caregivers for testing, treatment and care, and are deeply affected by social, structural and familial dynamics. Too often, health systems treat management of pregnancy and prevention and treatment of HIV as parallel rather than interconnected priorities, missing opportunities to intervene early in the lives of children born to mothers at risk for or living with HIV. Compounding these challenges are growing funding constraints, which threaten to reverse the progress achieved over the past two decades and jeopardize the sustainability of existing paediatric HIV programmes.

Over the past two decades, the paediatric HIV treatment landscape has evolved substantially. Treat‐all policies, expanded access to prevention of vertical transmission (PVT) services, routine maternal ART during pregnancy and breastfeeding, and the introduction of optimized paediatric ART formulations, including dolutegravir‐based regimens aligned across age and weight bands, represent major advances in paediatric care. These innovations have simplified treatment, improved tolerability and reduced regimen complexity compared to earlier paediatric formulations. However, despite these advances, persistent gaps in EID, linkage to care, retention and sustained viral suppression remain, particularly among infants and young children. Structural, caregiver‐dependent and service delivery model challenges identified in earlier eras continue to shape outcomes, underscoring the need for models that fully leverage contemporary treatment advances.

This commentary explores the opportunity to reimagine paediatric HIV care through integrated, evidence‐based child‐centred models that meet the unique needs of children, drawing from real‐world programmes and effective interventions. Spanning from mother–infant follow‐up strategies to advanced paediatric HIV disease management, we present evidence‐based models of care that show promise in closing gaps in diagnosis, treatment and long‐term retention in HIV care. Children must no longer remain on the margins of the HIV response if epidemic control is to be achieved.

### 1.1 Current Challenges in Paediatric HIV Diagnosis and Treatment

While many high‐burden countries have made strides in implementing vertical transmission prevention and EID, significant gaps persist, particularly in low‐burden regions and settings where health systems have historically devoted fewer resources to paediatric HIV. Globally, EID coverage remains well below universal. In 2023, only an estimated 67% of HIV‐exposed infants received a virologic test by 2 months of age, with coverage as low as 27% in western and central Africa, underscoring marked inequities in timely testing and follow‐up [2], even when mothers are engaged in care [3]. Delayed diagnosis and treatment initiation contribute to high mortality among infants who acquire HIV [4]. Without ART, nearly 50% of infants who acquire HIV will die by the age of 2 [5]. Timely EID, consistent follow‐up and repeat testing throughout the entire period of HIV exposure, including breastfeeding, are, therefore, critical.

For children who are diagnosed with HIV, further challenges arise. Linkage to treatment is often delayed or inconsistent, and retention in care remains weak. Structural barriers, including limited caregiver engagement, stigma and fragmented service delivery, impede continuity of care needed for viral suppression. Many health systems continue to rely on adult‐oriented models of care with minimal adaptation to the clinical, developmental and psychosocial needs of children and their caregivers [6].

Limited access to optimal paediatric ART formulations and point‐of‐care technologies for viral load monitoring further constrain good clinical outcomes for children. Gaps in routine viral load monitoring result in delayed or missed identification of children experiencing treatment failure. As a result, viral load testing and suppression rates among children remain significantly lower compared to adults [7]. However, the expanded use of optimized child‐friendly dolutegravir‐based regimens has the potential to significantly improve adherence and provides, therefore, a major opportunity to close the gaps in viral suppression in children [8].

Addressing these challenges requires models that embrace community‐based, family‐centred and differentiated models that are aligned with the complex needs of childhood and families affected by HIV. Integrating paediatric care into broader maternal and child health platforms can improve clinical outcomes while strengthening health systems [9].

## 2 Discussion: Centering Children in Models of Care: Promising Approaches

Persistent gaps in paediatric HIV diagnosis, treatment and retention underscore the urgent need to rethink how services are delivered for infants and young children. While barriers are well documented, innovative, child‐centred models that have demonstrated real‐world effectiveness have received less attention. We highlight promising approaches that illustrate how centring children in the HIV response can improve outcomes and strengthen systems of care. Table 1 summarizes these models of care for children.

**TABLE 1:** Summary of models of care.

Model of care	Key features	Example programmes/countries	Outcomes/impact
Integrated mother−infant follow‐up	Links antenatal, postnatal and paediatric services; supports both mother and infant throughout the HIV care cascade	IMPROVE in Lesotho; Integrated mother−infant clinic in Rwanda	Improved maternal ART adherence, viral suppression, EID rates and retention in care
Family‐centred care models (FCCMs)	Engages entire household in care; facilitates joint appointments, disclosure and adherence support	FCCM in Eswatini; Family clinic day in Uganda; Secondary caregiver support in Uganda; caregiver workshops: Yika Mpiko Model (DRC) Jitambu Model (Jitambu)	Improved viral suppression, family cohesion, appointment adherence and treatment support
Advanced HIV care strategies	Focuses on children with late presentation; uses paediatric DTG, CrAg screening and early adherence support, early diagnostics and management of advanced disease; tailored services for complex cases	DTG rollout in Mozambique; Paediatric advanced care clinic models in Kenya and Uganda; New Horizons Collaborative across multiple countries	Reduced paediatric mortality, improved health treatment outcomes and strengthened care for advanced HIV and treatment failure
Community‐based interventions	Delivers services in community settings via peer‐led adherence support, mentor mothers, lay health workers and stigma‐free entry points	PURE trial in Malawi; Red Carpet programme in Kenya; Community health worker programmes in Uganda; Hair salon model in Lesotho; community focal mothers in Eswatini	Increased ART retention and viral suppression, early diagnosis and stigma reduction

### 2.1 Integrated Mother−Infant Follow‐Up Models

Integrated mother−infant follow‐up models provide a critical link between prenatal and postnatal care, improving health outcomes among women and their infants, ensuring early diagnosis in infants exposed to HIV and expedited linkage to treatment for infants with HIV [10]. While gains have been made in initiating ART during pregnancy, sustaining engagement in care among women with HIV after delivery and during breastfeeding remains a challenge. In Rwanda, combined clinics for mothers with HIV and their infants demonstrated high retention, including 93% of infants completing follow‐up through final HIV status confirmation, with low HIV transmission rates (∼1.1%) [11]. Structured postnatal follow‐up models, such as the IMPROVE intervention in Lesotho, which included use of multidisciplinary care teams, enhanced training in patient‐centred HIV and maternal child health (MCH) care and early community‐based support, have demonstrated improved maternal ART adherence, viral suppression, contraceptive uptake and higher EID rates [12]. Facility‐based models are further strengthened by community‐based follow‐up, including home visits and engagement of lay health workers, which enhance retention and early clinical responses. Additionally, retesting women with negative HIV status in late pregnancy and during breastfeeding and the offer of pre‐exposure prophylaxis (PrEP) to women at risk have emerged as cost‐effective strategies to prevent vertical transmission, particularly among high‐risk populations [13, 14]. HIV self‐testing (HIVST) offers a complementary approach, expanding access to testing for pregnant women and their partners [15], as well as children in the household that may have been missed by PVT programmes. However, the HIVST strategy requires robust safeguards to ensure rapid and effective linkage to care. Taken together, these models underscore the importance of integrated, mother−infant dyad‐centred approaches in strengthening maternal and paediatric HIV outcomes.

### 2.2 Family‐Centred Models

Building on the importance of mother−infant dyad‐centred care, family‐centred models take a broader, holistic and practical approach by engaging the entire household to improve health outcomes among CLHIV. These models integrate services for parents/caregivers and siblings, recognizing the family as the unit of care. In Eswatini, a randomized control trial of a family‐centred care model showed modest but meaningful gains in viral load suppression among children when family members attended joint clinic visits [16]. Caregivers and providers reported additional benefits, including improved communication, stronger family bonds, easier disclosure and reduced clinic burden [12]. Similarly, Uganda's Family Clinic Day intervention, which synchronizes family appointments and offers peer‐led health education, was associated with significantly improved appointment adherence, an important precursor to long‐term retention and viral suppression [17]. Complementary evidence also points to the need for greater inclusion of fathers and male caregivers, particularly in adolescent parenting contexts, to improve maternal and child health outcomes [18].

In addition, secondary caregiver support models, designed to equip caregivers with the knowledge, tools and confidence to manage a child's treatment, are critical to ensuring consistent adherence, retention and overall wellbeing. These models recognize the central role caregivers play in a child's health, especially where children are dependent on adults to access and remain in care. In Uganda, models of care that identified and incorporated secondary caregivers into counselling and structured follow‐up of the child demonstrated improved viral suppression and retention in care [19]. In Tanzania and the Democratic Republic of the Congo (DRC), workshops led by multidisciplinary teams support caregivers in addressing the challenges their children face in adhering to treatment and achieving viral suppression, including guidance on disclosure [20, 21]. Despite their promise, family care models remain underutilized in many high‐burden settings, often sidelined in favour of fragmented, individual‐focused approaches. Embedding these strategies into routine HIV programming can significantly improve outcomes for children while enhancing the sustainability and equity of services across the HIV care continuum [22].

### 2.3 Advanced HIV Care Models

As paediatric HIV treatment continues to evolve, advanced care models are emerging to differentiate care for children not responding optimally to treatment, sustain long‐term viral suppression and improve survival. For children who are failing on dolutegravir‐based ART, critically important access to enhanced adherence counselling, HIV resistance testing and alternative treatment options remains suboptimal [23, 24]. Efforts in children have continued to lag [25]. Holistic, system‐level strategies are needed to close this gap. Innovations such as community‐based early adherence support, cryptococcal antigen (CrAg) testing and rapid diagnostic bundles (e.g. POC EID, same‐day confirmatory HIV testing, rapid TB screening), including opportunistic infections [26, 27, 28, 29, 30]. Ensuring these lifesaving approaches reach children will require deliberate investment, paediatric‐specific implementation research, and coordinated global action to prevent avoidable deaths and support children in achieving and sustaining viral suppression.

### 2.4 Community‐Based Approaches

Building on facility and family‐centred models, community‐based approaches are proving vital to extending the reach and effectiveness of paediatric HIV care, especially in contexts where health system access is constrained. Community‐driven interventions anchor services in trusted local actors to reduce stigma, improve access and support engagement [31]. In Lesotho, for example, hair salons offer young women stigma‐free entry points for accessing HIVST, PrEP and post‐exposure prophylaxis [32]. Peer‐based models such as Malawi's PVT Uptake and REtention (PURE) trial demonstrated significantly higher 2‐year ART retention among women receiving community support [33], while Kenya's Red‐Carpet programme leveraged engagement in care of adolescents newly diagnosed with HIV [34, 35]. Peer‐based and mentor−mother models have been shown to improve retention of PVT clients and their infants in care. In Kenya, a community‐based support programme for vulnerable pregnant adolescents and adolescent mothers used a home‐visitation, case‐management, team‐based approach to strengthen family and community support. This approach achieved high uptake of key interventions, including ART retention, EID, and increased rates of skilled birth attendance and family planning [36]. The Community Focal Mothers programme in Eswatini, where trained community volunteers remind mothers of clinic visits and emphasize the importance of continuing infant care, has successfully improved retention of mother−infant pairs in HIV services [37]. While multi‐month dispensing (MMD) models are designed for patients stable in care, evidence shows that consistent engagement in care remains critical. Among children enrolled in MMD, less frequent clinic visits may contribute to gaps in viral load monitoring or dosing revisions based on weight gain, highlighting the importance of investing in community structures and well‐supported cadres to bridge gaps in care for children and their families [38, 39].

## 3 Conclusions: The Way Forward and Call to Action

Over the past decade, the paediatric HIV treatment landscape has shifted substantially with the adoption of treat‐all policies, expansion of PVT services and introduction of optimized regimens. These advances have addressed many of the pharmacological and operational barriers that characterized earlier eras of paediatric treatment, when regimens were poorly tolerated, difficult to administer or unavailable. While treatment optimization has created new opportunities to improve paediatric outcomes, it has not been sufficient on its own to close the persistent gaps across the paediatric cascade. The progress and promise of paediatric HIV models—from family and facility‐based strategies to community‐driven innovations—demonstrate what is possible when children are intentionally centred in HIV programme design. Yet, these successes remain fragmented and underutilized. A critical gap continues to persist in scaling these effective models nationally and globally, especially in low‐resource settings where systems are often strained, and funding is increasingly uncertain.

We must move beyond pilot programmes towards nationally integrated, adequately resourced models of care. This requires political will, dedicated investment and intentional inclusion of children in HIV strategic planning at all levels. Priorities include stronger, integrated MCH platforms, family‐focused and community‐anchored support systems and removal of operational barriers. In addition, innovations in diagnostics, drug formulations and differentiated service delivery must be adapted to be accessible to children, not just adults. As service delivery models continue to evolve, emerging technologies such as artificial intelligence offer new opportunities to enhance predictive risk monitoring, personalize support and improve retention in care [40]. Ensuring these innovations are adapted for and inclusive of children will be essential to closing persistent gaps across the paediatric cascade.

The call to action is clear: global, national and local HIV responses must recognize children as a distinct population and an urgent priority—not an afterthought. A sustained, child‐centred approach, grounded in equity, community partnership and integrated systems is not just feasible, it is imperative. The path to HIV epidemic control cannot leave the youngest behind.

## Author Contributions

All authors discussed and conceptualized the manuscript. FT wrote the first draft of the manuscript. MM developed summary tables of published models of care. All authors contributed additional drafts and revisions of the manuscript.

## Funding

No specific funding was received to prepare this manuscript from funding agencies in public, commercial, or not‐for‐profit institutions or companies.

## Disclaimer

The views expressed in this manuscript are those of the authors and not necessarily of EGPAF nor even of any donor.

## Conflicts of Interest

All authors declare no conflicts of interest. The authors did not receive any support from any organization for the submitted work, nor even have financial relationships with any organizations that might have an interest in the current work submitted for publication.

## Data Availability

This analysis relies solely on publicly available data sources, including national reports and published datasets. No primary data were collected or generated for this brief.
